# Light-induced Nrf2^−/−^ mice as atrophic age-related macular degeneration model and treatment with nanoceria laden injectable hydrogel

**DOI:** 10.1038/s41598-019-51151-7

**Published:** 2019-10-10

**Authors:** Kai Wang, Min Zheng, Kaitlyn Lee Lester, Zongchao Han

**Affiliations:** 1 Department of Ophthalmology, the University of North Carolina at Chapel Hill, Chapel Hill, NC 27599 USA; 2 Carolina Institute for Nano Medicine, the University of North Carolina at Chapel Hill, Chapel Hill, NC 27599 USA; 3 Division of Pharmacoengineering & Molecular Pharmaceutics, Eshelman School of Pharmacy, the University of North Carolina at Chapel Hill, Chapel Hill, NC 27599 USA

**Keywords:** Biotechnology, Diseases

## Abstract

Elevated oxidative stress and associated reactive oxygen species (ROS) accumulation are hallmarks in the induction and progression of age-related macular degeneration (AMD). By exposing nuclear factor erythroid 2-related factor (Nrf2) knockout (Nrf2^−/−^) mice to mild white light, we were able to generate a new dry-AMD like murine model to the study. This animal model developed phenotypes of photoreceptor degeneration, retinal function impairment, ROS accumulation, and inflammation reaction in a relatively shorter time. In the treatment of this animal model we utilized an antioxidative and water soluble nanoparticle known as glycol chitosan coated cerium oxide nanoparticles (GCCNP). The delivery of GCCNP protected retina against progressive retinal oxidative damage. Further combination of GCCNP with alginate-gelatin based injectable hydrogel provided synergistic antioxidant effects and achieved a more rapid recovery of the retinal pigment epithelium and photoreceptor cells. This combined treatment technique has the potential to translate into a clinical intervention for the treatment of AMD.

## Introduction

Age-related macular degeneration (AMD) is one of the leading causes of legal blindness among the geriatric age class, affecting an estimated ninety-six million people worldwide^1–4^. AMD can be characterized by the presence of drusen, accumulation of debris within Bruch’s membrane (BrM), atrophy of the retinal pigment epithelium (RPE) and photoreceptor cells (PRC), or neovascularization. Approximately 90% of patients progressed to atrophic (dry) AMD with severe vision impairment, the remaining 10% caused neovascular (wet) AMD and led to major legal blindness^5^.

The cause of AMD remains elusive, but several risk factors (e. g. aging, smoking, hypertension, high body weight index, chronic inflammation, direct exposure to sun light, and/or lacking of dietary antioxidant) were determined in clinical studies^6–8^. All the risk factors are directly or indirectly associated with elevated reactive oxygen species (ROS) in RPE and PRC cells^9^. Several signaling studies revealed dysfunction of the antioxidation responses and/or ROS related to AMD^10^. Although there are no current FDA-approved drugs for dry AMD, a major clinical trial sponsored by NEI, tested the effects of high dosage dietary antioxidative supplements on individuals with dry AMD. The major supplements of this Age-Related Eye Disease Study (AREDS) included: vitamin C/E, zinc, copper, and β-carotene. Newer formulations (AREDS2) replace the β-carotene in the original AREDS formula, with lutein/zeaxanthin. This new formula (AREDS2) is now used as the first-line treatment for dry AMD. Although these supplements showed a ~25% beneficial effect in reducing the risk of progression towards advanced dry AMD, the extremely short half-life, weak activity, and correlated cancer risks of the high dosage of supplements have shown mix results^10,11^. Although there are several potential AMD medications under clinical trials, new medicine trials rarely proceeded to phase 3 dues to limited treatment effect. The neuroprotectants (Tandospirone or NT-501) ended at Phase 2 of the clinical trials because they were not able to stop the progression of dry AMD^12,13^. The complement factor C5 inhibitor Eculizumab also failed at phase 2 due to the failure in slowing geographic atrophy (GA) and drusen volume increase, and Lampalizumab (Anti-Factor D) progressed to phase 3 clinical but unfortunately showed identical results to untreated groups^12,14^. The b-amyloid antibody and drug, which removes lipids or accumulated oxidative waste, did not contribute to AMD treatment. Thus, the treatment of atrophic AMD should not involve merely one factor, but instead should be comprised of multiple factors which target to remove oxidative waste, protect RPE and PRC, and suppress inflammation, leading to a more promising approach to AMD treatment.

A long-term antioxidant will be highly preferable in AMD treatment. Nanoceria is a rare earth oxide, which has shown robust antioxidative activities in various biomedical applications to scavenge ROS due to its auto-regeneration properties – to shift oxidation between its two oxidation state (Ce^3+^/Ce^4+^)^15,16^. Nanoceria improved cell survival and proliferation by reducing ROS in the microenvironment in several studies^15,17,18^. The intravitreal injection of nanoceria protected rat retinal function from light damage and reduced the inflammatory response of the vldlr^−/−^ murine model^19^. Over the duration of a year, nanoceria was able to retain antioxidative properties. Recently, we developed a water-soluble form of nanoceria, known as glycol chitosan coated cerium oxide nanoparticles (GCCNP). We found the antioxidant activity of GCCNP were significantly higher than the uncoated nanoceria^16^. Additionally, the combination of GCCNP with injectable hydrogels showed further controlled delivery and treatment efficacy^16,20^. These results suggested that the self-gelling systems will provide therapeutic synergy to AMD for determining its long-term and sustained therapeutic benefits. Compared to other drug delivery methods for AMD treatment, which are still in clinical trials (e.g. Portable Delivery System from Genentech, phase 2 and Encapsulated Cell Therapy from Neurotech, phase 2), injectable hydrogels do not require a surgical procedure for their implantation and removal, further making injectable hydrogel a desirable replacement of implanted drug delivery devices.

Due to lack of macular in the retina, there are no ideal rodent models that truly mimic human AMD phenotypes^21,22^. Nrf2^−/−^ mice have atrophic AMD phenotypes such as drusen like deposits, GA, inflammation, and retinal function impairment^23^. However, those knock out murine models require at least 12 months to develop the desired phenotypes, which makes the treatment hard to conduct. Studies have shown that the combination of two or more AMD risk factors has the potential to create a desired animal model in a relatively short time. For instance, blue light and UV light can directly contribute to degeneration of RPE and PRC, and the combination of long-term light exposure and deficiency in SOD1 gene expression generated dry AMD phenotypes^24,25^. MEF2D^−/−^ mice that downregulate the expression of the antioxidative response Nrf2 gene, are susceptible to retinal light damage and light-induced PRC damaged phenotypes, which can be generated with less light intensity (~4000 lux) rather than commonly used 10000–13000 lux on pigmented mice^26^.

We introduced a new murine dry AMD-like model using Nrf2^−/−^ mice combined with light exposure-induced damage. Our results showed that the AMD-like retinal pathology of Nrf2^−/−^ was triggered by a 3-h mild white light exposure (4,500 lux) at 2 months of age and resulted in permanent retinal functional loss, atrophy of RPE and PRC, drusen-like-deposits, and an inflammatory response. Furthermore, the treatment using GCCNP-laden hydrogels allows for sustained release of antioxidative GCCNP and renders synergistic antioxidative effects on PRC and RPE cells. This treatment is highly likely to protect RPE and PRC from further oxidative damage while enhancing their restoration, thus having the potential to translate to the clinical field.

## Result

### Nrf2^−/−^ mice are susceptible to light-induced retinal lesions

**Figure 1:**
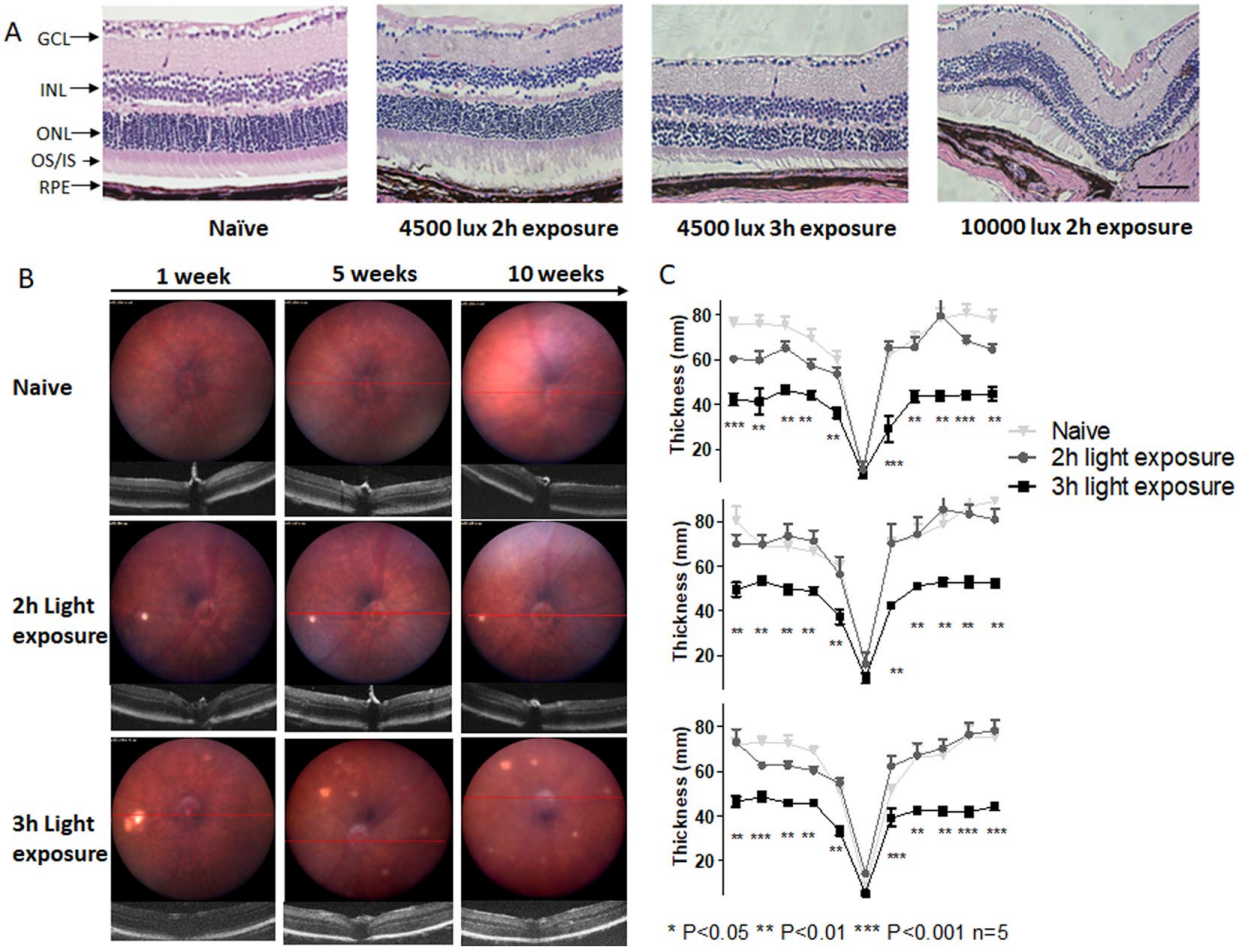
Mild light exposure can induce Nrf2^−/−^ mice retina damage. (**A**) Paraffin H&E histology studies exhibited damage from 2 h 4500 lux light exposure, irreversible damage from 3 h 4500 lux damage, more severe damage from 2 h 10000 lux damage. Scale Bar 50 µm. (**B**) The Fundus and OCT images of Nrf2^−/−^ mice. Naïve, 2 h, and 3 h 4500 lux light exposure groups. (**C**) The retina ONL thickness (n = 5) measured by OCT and averaged thickness of retina ONL in all the groups by 1, 5, and 10 weeks. The 3 h light exposure caused reduction in ONL thickness. Distance from ONH: 1.7 mm. (One-way ANOVA with Tukey’s multiple comparison test, error bars indicate SEM n = 5, *P < 0.05, **P < 0.01, ***P < 0.001).

Study has shown that the downregulation of Nrf2 expression leads to compromised regulation of oxidative stress, leaving the retina more prone to light induced oxidative damage^26^. To examine whether light exposure can lead to PRC or RPE cells atrophy, we exposed Nrf2^−/−^ mice on different intensity of light exposure at 4,500 lux for 2 h and 3 h, and 10,000 lux at 2 h. We found that Nrf2^−/−^ mice can be triggered by a 3 h, mild white light exposure (4,500 lux) at 2 months of age (P 2 m), resulting in permanent PRC/RPE impairment. At 10 weeks post-3 h exposure, histological studies showed that the outer nuclear layer (ONL) of the retina was thinned and nearly 50% PRC nuclei were absent after 3 h light exposure, indicating irreversible PRC degeneration. The structure of OS/IS were also found to be disrupted after light exposure (Fig. 1A). 2 h light exposure showed that ONL thickness appeared normal. The 10000 lux 2 h damage caused the most severe damage and the majority of ONL degenerated at the endpoint. The evident sign of damage or degeneration was absent from INL and GCL from histological sections, suggesting the major ROS elevation mainly exist in light-sensitive PRC.

The Funduscopy revealed retinal morphological changes after exposure to light (Fig. 1B). Apparently, 4500 lux 2 h light exposure caused lesions on the retina at 1 week post-2 h light exposure, however the lesions were recovered 10 weeks post exposure. 3 h light exposure induced more severe lesions as revealed by the increased accumulation of bright-yellow spot, and more lesion spots became visible with time passing. We measured the thickness change of the ONL post exposure by optical coherence tomography (OCT), and 3 h 4500 lux light could lead to GA-like phenotypes, such as degeneration of PRC and RPE. The naïve Nrf2^−/−^ mice have an average ONL thickness of approximately 75 µm at 6 weeks post-natal (Fig. 1C). 2 h 4,500 lux caused a ~20% reduction of ONL thickness (60 ± 10 µm) at the first week-posted light exposure, but the thickness of ONL was found recovered at the fifth week post light exposure (70 ± 8 µm). 3 h light exposure group had more severe damage than 2 h exposure at 4,500 lux on the first week (41 ± 5 µm, ~50% reduction in ONL thickness compared to naïve mice, P = 0.013). Although there was partial recovery after five weeks, the degeneration of ONL thickness could not be reversed (49 ± 6 µm, ~40% ONL thickness reduction compared to naïve mice, P = 0.004). The 2 h 10000 lux resulted in severe cataracts after light exposure, creating challenges to perform Fundus, OCT, and electroretinography (ERG). Therefore, we choose 4500 lux 3 h light exposure for further studies, where the damage mainly existed on ONL and RPE (Fig. 1A,B).

### The light-induced Nrf2^−/−^ mice have dry AMD-like retinal pathology

**Figure 2:**
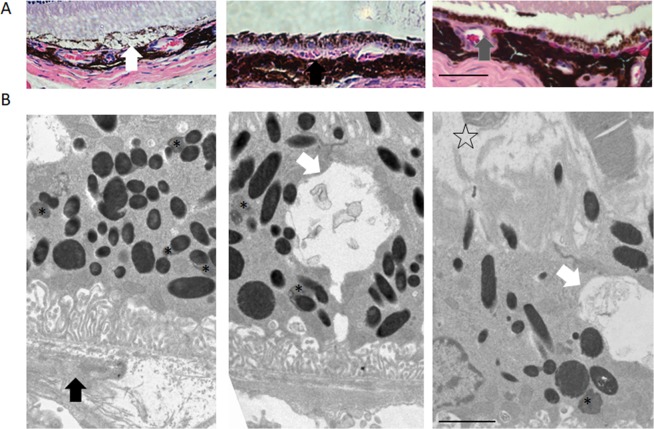
Nrf2^−/−^ mice can develop atrophic AMD-like phenotypes after mild light exposure. (**A**) The H&E histology showed the dry AMD phenotypes on light exposure induced Nrf2^−/−^ model. From left to right: vacuole, drusen-like deposit, and choroid vascular changes, Scale Bar 50 µm. (**B**) The TEM images showed the dry AMD phenotypes on light damaged Nrf2^−/−^ model. (White Arrow: vacuole, black arrow: drusen-like deposit, asterisk: lipofuscin granule, and star-shape: PRC cells detachment). Scale bar: 2 µm. Original images were presented in Supplementary Fig. S6.

To further examine whether the light-induced Nrf2^−/−^ mice have dry AMD-like retinal pathology, we performed fundus/OCT imaging, histology, TEM, and ERG analyses. We found that bright white-yellowish spots appeared on the Fundus images in the most light-exposed eyes (41 out of 50). OCT images suggested a drusen-like structures, which are located under RPE, disrupted the PRC and RPE structure. Retinal histology analysis identified drusen-like alterations of RPE as shown in Fig. 2A. However, most of the deposit under RPE was found in a basal linear formation, rather than a granular-shaped drusen as shown by others in advanced stage atrophic AMD^27^ (Fig. 2A). Other morphologic alterations include hemorrhage and vacuole, indicating the accumulated toxic products and cytokines responsible for RPE degeneration (Fig. 2A). In addition, capillary changes can be found between choroid and RPE (Fig. 2A), but there is no clear evidence that this model can generate choroidal neovascularization at the endpoint of experiments. TEM images displayed the accumulated autophagosome and lipofuscin in RPE (Fig. 2B) and a significant increase of the BrM thickness (1.12 ± 0.25 µm, P = 0.005) compared with un-treated controls (Fig. 2B). The detachment of PRC from RPE was also observed from TEM images.

Funduscopy revealed excess concentrations of oxidized lipoprotein in RPE at 4-month post light exposure groups. (Supplementary Materials S1). Lipofuscin can be detected using autofluorescence from 488 nm light source excitation. Study showed that lipofuscin/drusen can generate intense green fluorescence (Supplementary Materials S2). The hemorrhage under RPE was also observed on histology samples. Additionally, the random localized lesion and thickening of choroid layer was also found on 3 h light exposed mice PI-10 weeks (Supplementary Materials S3).

**Figure 3:**
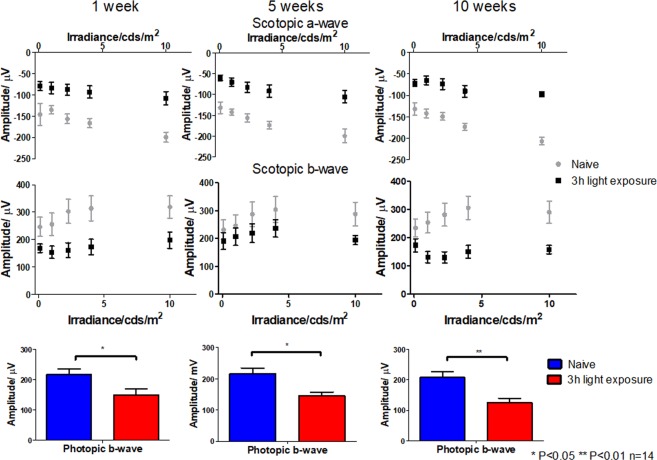
Light exposure compromised Nrf2^−/−^ mice retinal function. Scotopic a-wave, scoptopic b-wave, and photopic b-wave ERG of Nrf2^−/−^ mice (n = 14). Naïve, 3 h 4500 lux light-exposed mice (One-way ANOVA with Tukey’s multiple comparison test, n = 14, error bars indicate SEM *P < 0.05, **P < 0.01, ***P < 0.001).

The ERG showed a significant reduced amplitude in PRC (Fig. 3). The scotopic a-wave represents the electric response from rod PRC cells, which are sensitive to dimmed light. The Nrf2^−/−^ mice loss approximately 50% response to lights (−107 ± 16 µV at 10 cd*s/m^2^) of the scotopic a-wave in the first week after light exposure. Although the response was slightly recovered after five weeks, the PRC function was further compromised at the endpoint (−97 ± 13 µV at 10 cd*s/m^2^, scotopic a-wave). These results are directly correlated to the PRC thickness change results, indicating the loss of PRC functionality. The photopic b-wave readings indicate the response from cone PRC cells. Similar to scotopic a-wave, the impairment of cone cells was significantly reduced (P = 0.017 1-week, P = 0.010 5-week, and P = 0.004 10-week) compared to naïve mice. The overall function of the retina (mainly from bipolar cells) from the scotopic b-wave also reduced ~33% in 1 week post light exposure (−149 ± 19 µV at 2.25 cd*s/m^2^). Although there were some recoveries after 5 weeks, the overall function of the retina was disrupted (−125 ± 18 µV at 2.25 cd*s/m^2^, ~40% reduction) after 10 weeks as direct result of accumulated ROS.

### The GCCNP laden hydrogel is biocompatible and non-toxic

Our previous research not only demonstrated the biocompatibility of GCCNP and injectable hydrogel, but also assessed the wide safe dose range in which GCCNP can be used^28^. In addition, delivery of nanoceria to ARPE-19 cells via injectable hydrogel was evaluated in our study and showed that hydrogels were biocompatible and sustained nanoceria release for a duration of two months without causing any side-effect. From Fundus and OCT images of Nrf2^−/−^ mice with the absence of light damage, the hydrogels started to degrade from the first-week post-injection and lost some content by 5 weeks of injection. By the time of 10 weeks, the residue of hydrogel was absent from the image and the hydrogels were degraded (Supplementary Materials S4). Compared to naïve Nrf2^−/−^ mice, there is no significant sign of inflammation response or toxicity to PRC or RPE. We assume that the sustained release character of the hydrogel prevented the dose dumping of GCCNP upon injection, assisting GCCNP in reaching the RPE in a sustained manner. From the fundus and OCT images of blank and GCCNP laden hydrogels intravitreal injection, properly injected hydrogel is clear, and does not cause cataracts and other damage to the anterior segment (Supplementary Materials S4). There is no evident signs of retinal structure changes or abnormalities to PRC or RPE as shown from Fundus and OCT. The retina retained a healthy formation and no retinal detachment or cataracts were found after injection.

### The GCCNP hydrogel protected RPE and PRC from structural deformation in light-induced Nrf2^−/−^ mice

**Figure 4:**
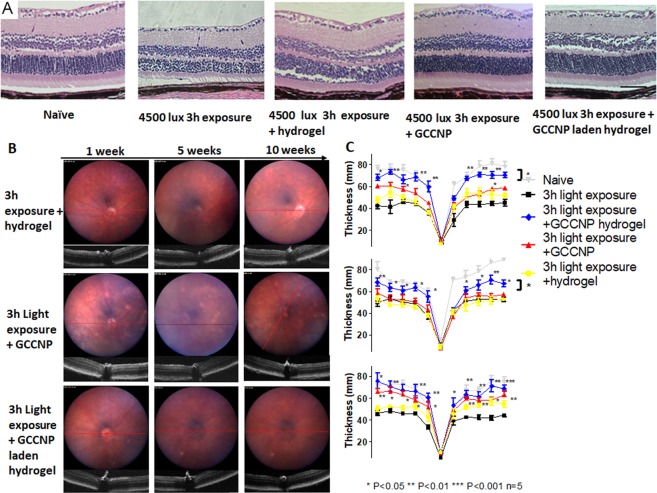
GCCNP and GCCNP laden hydrogel provide structurally rescue in light-induced Nrf2^−/−^ mice retina degeneration. (**A**) Paraffin H&E histology studies exhibited 3 h 4500 lux damage treated by GCCNP and GCCNP laden hydrogel. (**B**) The Fundus and OCT images of Nrf2^−/−^ mice. 3 h 4500 lux light exposure groups treated by 1 µg GCCNP, 1 µL hydrogel, or 1 µg GCCNP in 1 µL hydrogel by intravitreal injection. (**C**) The retina ONL thickness (n = 5) measured by OCT and averaged thickness of retina ONL in all the groups by 1, 5, and 10 weeks. ONL thickness was rescued with GCCNP with/without hydrogel. Distance from ONH: 1.7 mm. (One-way ANOVA with Tukey’s multiple comparison test, error bars indicate SEM n = 5, *P < 0.05, **P < 0.01, ***P < 0.001).

To investigate whether GCCNP and its injectable hydrogel can improve the morphological changes in the light-induced Nrf2^−/−^ mice model’s RPE and PRC, we injected GCCNP and the injectable hydrogel (with or without GCCNP) to 3-day post light exposure Nrf2^−/−^ mice. No adverse events or inflammatory reactions were observed post-injection. At 10 weeks post-injection (PI), histological staining showed that standalone hydrogel didn’t make any progress on RPE abnormality, but partially improved PRC recovery, although not reaching significance. In light induced Nrf2^−/−^ mice, both GCCNP and GCCNP laden hydrogel groups exhibited regeneration of PRC structure and morphology. These two groups also displayed an ONL thickness comparable to naïve Nrf2^−/−^ mice on both PRC and RPE (Fig. 4A).

From Fundus/OCT scans, the injectable hydrogel only group did not show any adverse events. At 1-week PI, no significant difference in ONL thickness was observed in GCCNP (60 ± 6 µm), hydrogel only (52 ± 10 µm), and GCCNP laden hydrogel (67 ± 7 µm) groups, compared to light-exposed group; although GCCNP laden hydrogel group achieved more notable ONL recovery as revealed by OCT measurement. The retina started to recover from PI-5 weeks in GCCNP hydrogel group (70 ± 10 µm) and lasting until the experiment endpoint (Fig. 4B). We did not find any obvious retinal thickness changes in the GCCNP standalone group until PI-10 weeks (58 ± 9 µm). The GCCNP hydrogel group showed significantly faster recovery compared to the GCCNP group (P = 0.049).

### The GCCNP hydrogel reduced AMD-like atrophy in light-induce Nrf2^−/−^ mice

From the histology sections, results showed that both GCCNP and GCCNP hydrogel treatments improved the AMD-like phenotypes in light-exposed Nrf2^−/−^ mice. Apparent granule/basal linear drusen, vacuole, hyper/hypo-pigmentation, or neovascularization was not observed from those mice (Fig. 4).

**Figure 5:**
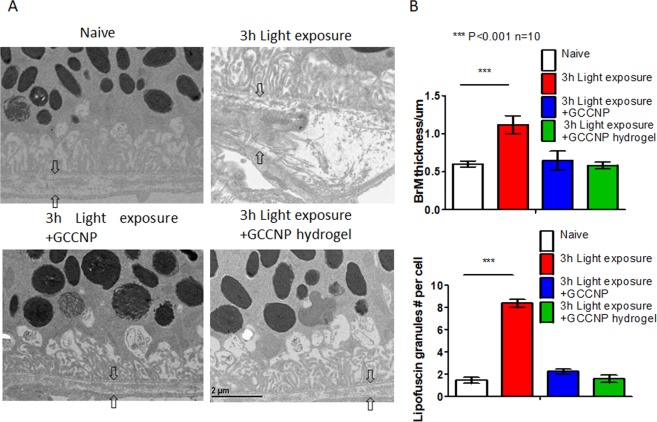
GCCNP and GCCNP laden hydrogel reduced AMD-like phenotypes on Nrf2^−/−^ mice. (**A**) TEM measured thickness of Bruch’s membrane. The light exposure caused irregular thicken BrM and GCCNP treated the thicken BrM. (**B**) The statistics of BrM thickness and lipofuscin granules number (One-way ANOVA, ***P < 0.001, n = 10).

The treatment using GCCNP and GCCNP hydrogel reduced the number of lipofuscin to the normal level (approximately 1–2 lipofuscin granules per cell), and the difference between treated groups and untreated groups is significant (P = 0.0005) (Fig. 5B). Compared to the untreated group, both GCCNP and GCCNP hydrogel achieved significant improvements (P < 0.0001). The BrMs of both treated mice were comparable to naïve eyes after the treatment (0.58–0.64 µm) (Fig. 5B). The electron-dense nanoceria was also observed in RPE cells with homogeneous distribution (Fig. 5A), as similar shown in our previous research and Supplementary Materials S3. From Fundus images of light-induced mice treated by GCCNP laden hydrogels, the green signal of lipofuscin was eliminated after the treatment (Supplemental Materials S2).

### Hydrogel-mediated GCCNP delivery rescued retinal functions in light-exposed Nrf2^−/−^ mice

**Figure 6:**
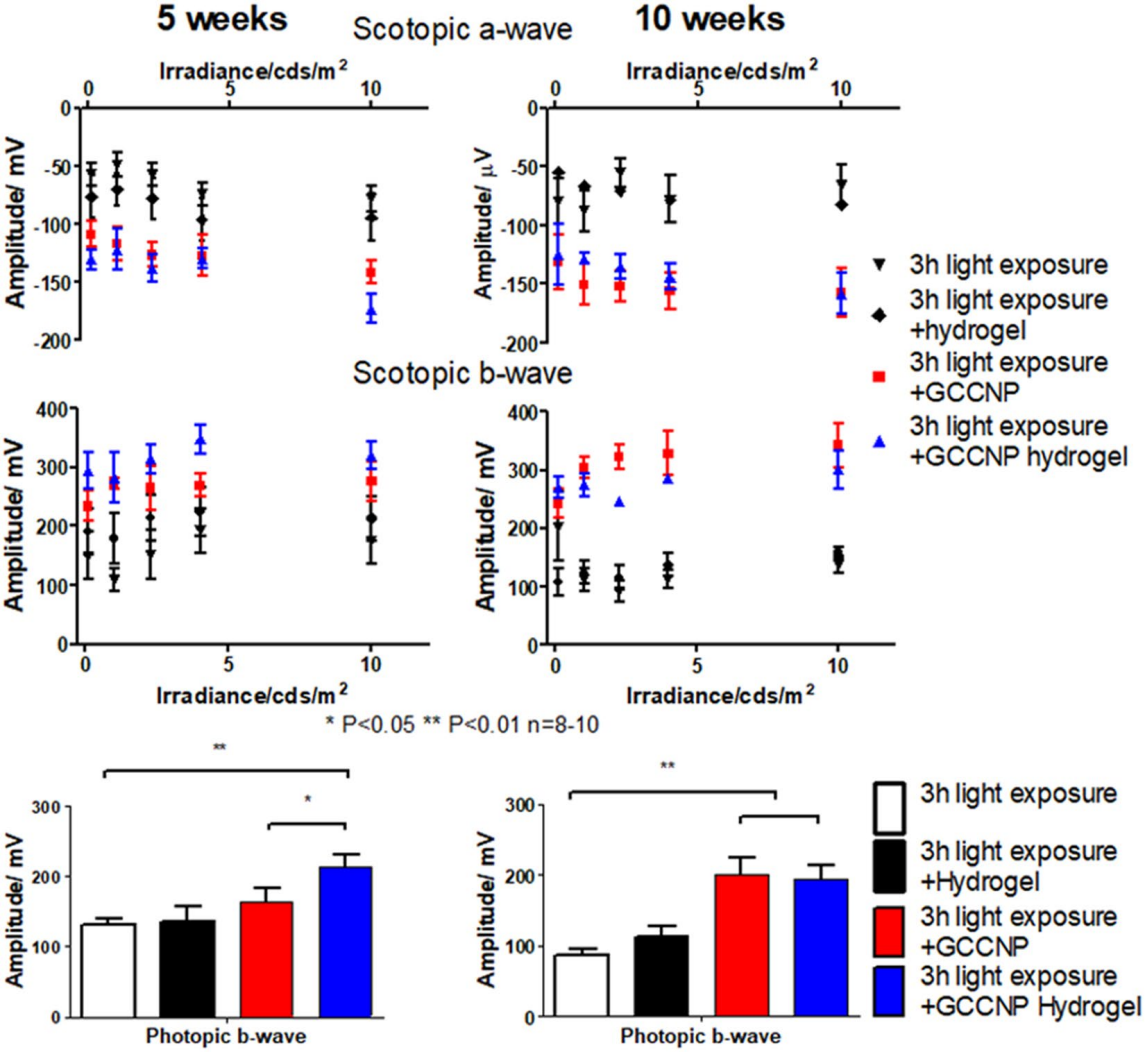
GCCNP laden hydrogel provides functional rescue in light-induce Nrf2^−/−^ mice retina. Scotopic a-wave, scotopic b-wave, and photopic b-wave ERG of 3 h 4500 lux light-exposed mice treated by 1 µg GCCNP, 1 µL hydrogel, or 1 µg GCCNP in 1 µL hydrogel by intravitreal injection (One-way ANOVA with Tukey’s multiple comparison test, error bars indicate SEM, n = 8–10, *P < 0.05, **P < 0.01, ***P < 0.001).

To determine whether GCCNP and GCCNP laden hydrogels can restore vision function in light-exposed Nrf2^−/−^ mice, ERGs were performed at PI-5 weeks and PI-10 weeks. When comparing GCCNP laden hydrogel mice to the results of standalone GCCNP treated mice (scotopic a wave and photopic b wave, Fig. 6) the difference in ERG retinal functions were significant (P = 0.047). GCCNP laden hydrogel groups showed earlier recovery periods, which could be facilitated by the GCCNP laden hydrogel treatment both scotopic (−173 ± 23 µV at 10 cd s/m^2^ scotopic a-wave) and photopic (213 ± 13 µV at 2.25 cd s/m^2^, photopic b-wave) ERGs at PI-5 weeks. At PI-10 weeks, the ERG signal from GCCNP and GCCNP hydrogel treated eyes reached a similar level (Fig. 6). Compared with untreated and standalone GCCNP treated mice, both treatments achieved statistically significant changes in the ERG retinal functions.

### GCCNP laden hydrogels prevented production of excessive ROS and pro-inflammatory protein in light-exposed Nrf2^−/−^ mice

**Figure 7:**
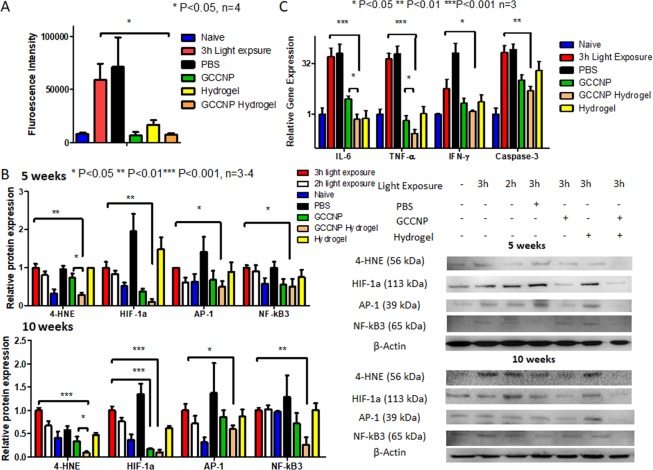
GCCNP laden hydrogel reduces oxidative stress and cytokines production in light-induced Nrf2^−/−^ mice eye. (**A**) DCF-DA assay result showed elevation of (**B**) Western blotting of 4-HNE, HIF-1α, AP-1, and NF-κB3 of Naïve, 2 h and 3 h 4500 lux light-exposed, 3 h 4500 lux light-exposed Nrf2^−/−^ mice (n = 3–4) treated by PBS, 1 µg GCCNP, or 1 µg GCCNP in 1 µL hydrogel by intravitreal injection at 5 weeks and 10 weeks. The product 4-HNE, HIF-1α, AP-1, and NF-κB3 were significiantly reduced in treated groups using GCCNP or GCCNP laden hydrogel PI-5 weeks or PI-10 weeks. (**C**) RT-PCR of Naïve, 2 h and 3 h 4500 lux light-exposed, 3 h 4500 lux light-exposed Nrf2^−/−^ mice (n = 3) treated by PBS, 1 µg GCCNP, or 1 µg GCCNP in 1 µL hydrogel by intravitreal injection at 10 weeks. The IL-6, TNF-α, and IFN-γ were determined. Cytokines production were reduced in treated groups using GCCNP alone or in hydrogel. Full-length blots are presented in Supplementary Fig. S7. (One-way ANOVA with Tukey’s multiple comparison test, error bars indicate SEM n = 3–4, *P < 0.05, **P < 0.01, ***P < 0.001).

Additionally, we investigated potential antioxidative properties of GCCNP and its injectable hydrogels using free radical assay, real-time Polymerase chain reaction (RT-PCR) and Western blotting. To directly asses the ROS quenching in PRC and RPE, we conducted dichlorodihydrofluorescein diacetate (DCF-DA) assay and luminol assay (Fig. 7A and Supplementary Materials Table 1). We found a substantial increase of fluorescence and luminescence signal in light-exposed Nrf2^−/−^ mice. Treatment with GCCNP and/or GCCNP injectable hydrogels significantly reduced the luminescence signal intensity to a close to normal value at PI-10 weeks (P = 0.012, n = 4).

Western blotting revealed that the light exposure resulted in an accumulation of oxidative products of 4-hydroxynonenal (HNE), cellular response to hypoxia product of hypoxia-inducible factor (HIF)-1a), and products of AP-1 and NF-κB3 (p65) proteins that involved in cell apoptosis and inflammation^9,29^. From Fig. 7B, the light-exposed retina produced a large amount of 4-HNE and HIF-1α at 5 and 10 weeks after light exposure, and those can be significantly reduced by the treatment of GCCNP and its injectable hydrogels. The GCCNP and hydrogel showed synergistic effects in protein studies (P < 0.001). In comparison with the GCCNP treated mice, hydrogels with GCCNP further reduced the production of 4-HNE (P = 0.0453 at PI-5 weeks and P = 0.0245 at PI-10 weeks). AP-1 and NF-κB3 were also significantly downregulated at PI-5 weeks (P = 0.017 AP-1 and P = 0.045 NF-κB3) and PI-10 weeks (P = 0.013 AP-1 and P = 0.007 NF-κB3) with GCCNP laden hydrogel.

Furthermore, our RT-PCR data showed that treatment of both GCCNP and its injectable hydrogels significantly reduced pro-inflammatory cytokines mRNA, i.e., interleukin (IL)-6, tumor necrosis factor (TNF) -α, interferon (IFN)-γ, and apoptosis-related protease caspase-3 compared to naïve Nrf2^−/−^ mice (Fig. 7C).

### Synergistic antioxidative mechanism of GCCNP and injectable hydrogel

To study the synergistic therapy mechanism of GCCNP and hydrogel, we performed the analysis of structural and dimensional changes of GCCNP released from hydrogel and compared with standalone GCCNP. The UV-Vis spectra showed absorption maximum of GCCNP at 295 nm and alginate-gelatin hydrogel at 260 nm, and the spectrum of GCCNP released from hydrogel combined maximum absorption values of both standalone GCCNP and alginate-gelatin hydrogel. It is hypothesized that chitosan and alginate formed polyplexes after hydrogel degradation^30^. To prove the hypothesis, GCCNP released from hydrogel and standalone GCCNP were imaged using TEM. It is clearly that GCCNP released from hydrogel has a much larger dimension compared to standalone GCCNP. The DLS size of standalone GCCNP is ~170 nm after sonication, but degraded hydrogel released GCCNP have a wider range of size 200–900 nm (Supplementary Materials S5). The zeta potential test showed opposite potential of alginate and standalone GCCNP. The alginate-gelatin hydrogel has ~−43 mV potential but GCCNP has over 30 mV potential. The released GCCNP reached ~−30 mV potential, which is the evidence that glycol chitosan and alginate formed polyplex and increased in size.

## Discussion

In human atrophic AMD, the antioxidative gene, Nrf2’s, expression is downregulated or compromised due to aging^31^. Deficiency or downregulation of Nrf2 gene expression led to failure in neutralization of excessive ROS and caused long-term oxidative stress on PRC and RPE cells^24,26^, thus the PRC and RPE cells will suffer from chronic oxidative stress related complication. Accumulated oxidized waste will accumulate and immunity (pro-inflammatory proteins, macrophages, and multiple complement factors) will be activated in response to both rising ROS and waste presence. Eventually, apoptosis and membrane attack complexes response will be triggered in the RPE and PRC cells, this cellular damage can result in higher order complications, including the loss of retinal function and inflammation.

Nrf2 is a transcription factor that regulates the expression of antioxidant proteins which serve to protect against oxidative damage triggered by injury and inflammation^10^. Nrf2^−/−^ mice have shown AMD-like retinal pathology including drusen-like deposits and RPE/BrM degeneration at their late age (12 months)^23^. In our newly generated murine model, we have shown that the AMD-like retinal pathology can be induced by a mild white light exposure in Nrf2^−/−^. Compared to aged (12 months) Nrf2^−/−^ AMD-like mouse model, this model saves valuable time and allows for easier access in the evaluation of atrophic AMD treatment. AMD pathology can be triggered by a 3 h, mild white light exposure (4500 lux) in lieu of commonly studied 10,000 lux at 2 months of age and result in permanent retinal functional loss. Visible light with a wavelength shorter than 470 nm may cause acute damage to RPE and PRC cells through elevation of ROS^24^. With the absence of antioxidative Nrf2 pathway activation, the oxidative stress in the Nrf2 gene knockout model is difficult to neutralize. Thus, the light exposure could trigger localized oxidative stress, resulting in the oxidation of lipids and accumulation of inflammatory proteins/nucleic acids.

The raising ROS levels can result in a wide range of vision impairment-related complications^23,26^, including inflammation and angiogenesis. The large vacuole of autophagy, which remove oxidized proteins/lipids, is a significant symbol of dry AMD, and this ROS driven process is minimized with GCCNP treatment^23^. The largely produced pro-inflammatory proteins, (HIF-1α, IL-6, and TNF-α) caused by ROS elevations, are determined from this murine model through multiple experiments, and can be well controlled under antioxidant treatment^32,33^. Moreover, our model has the potential to develop wet AMD-like phenotypes. In a 4-month time period our murine model exhibited abnormal changes among choroidal capillaries, which is an indicative sign of neovascularization.

The sustained and auto-regenerative antioxidative properties of GCCNP were evaluated in our previous publication. Preliminary studies using GCCNP and alginate-gelatin hydrogel on ARPE-19 cell lines proved GCCNP’s anti-oxidation and anti-inflammation feasibility and determined the safe dosage range for injection^16,28^. The treatment using GCCNP rescued both structure and function of the retina. The combined treatment of the alginate-gelatin injectable hydrogel with GCCNP was able to achieve a rapid recovery of RPE and PRC within 5 weeks. This combined treatment proved to be superior to the standalone GCCNP which took up to 10 weeks for results of the treatment to prevail. Previous studies displayed not only the robust synergistic antioxidative properties of alginate-gelatin injectable hydrogel, but also its ability to reduced inflammation and angiogenesis^28^. As a sustained releasing vehicle, the hydrogel prevented initial burst release of GCCNP, reducing complication of GCCNP overdose (e.g. cytotoxicity) and allowing for a more homogeneous distribution under the retina. After mixing with hydrogel, GCCNP formed polyplexes with alginate moiety of hydrogel, and trafficking of GCCNP allowed alginate to enter the PRC and RPE cells. The negative charge of the alginate moiety of the polyplex is believed to aid in the transportation of GCCNP^34^. The alginate moiety can also contributes to the scavenging of free radicals^35^. Therefore, GCCNP facilitates the transportation of alginate and performs synergistic antioxidation.

As an effect of the alginate treatment, PRC were shown to have an earlier recovery compared to standalone GCCNP treatment from ERG and WB study. In addition, the efficacy of GCCNP in wet AMD treatment was shown in separate anti-angiogenesis studies using laser damaged murine models^16^.

Thus, the combination of GCCNP and injectable hydrogel is highly promising for AMD treatment, and proposes a feasible treatment plan for other elevated oxidative stress associated ophthalmic diseases (e.g. diabetic retinopathy)^36^. However, the mismatch of fast novel hydrogel formulation development and rigorous FDA approval procedure, prevents most hydrogels from being approved in a drug delivery purpose. Furthermore, the long-term safety and effectiveness of nanoceria still requires further studies and may not be available for clinical trials in the near future. Long-term evaluation of GCCNP and hydrogel are still requested before clinical trial on dry AMD patients.

## Conclusion

Nrf2^−/−^ mice can generate the phenotype of dry AMD after moderate light exposure at their early age. These phenotypes included inflammation, drusen-like formation, compromised retinal function, degeneration of PRC and RPE, and accumulation of oxidized products. This model saved time and displays similar phenotypes to that of their aged counterparts, providing new tools to study dry-AMD drug intervention. GCCNP delivery via intravitreal injection reduced ROS caused damage in the mouse retina. The GCCNP laden hydrogel further improves the treatment’s efficacy compared to standalone GCCNP. The combination of GCCNP and injectable hydrogel have potential to translate to the clinical field for treatment of dry AMD.

## Methods

### Hydrogel and GCCNP Synthesis

The hydrogel and GCCNP synthesis was performed according to our previous research^16,20,28^. Briefly, 0.7 M cerium chloride was added to 2% glycol chitosan solution and stir at ambient temperature. Then 28–30% ammonium hydroxide was added and stirred at 5000 rpm for 16 h. Supernatant was collected and dialyzed against Millipore water (20 kDa MW) to obtain nanoceria with glycol chitosan coating. 5 mL 2% alginate aqueous solution mixed with 5 mL 2% gelatin aqueous solution in 20 mL vial. Next, 40 mg EDC and 30 mg NHS was added and reacted at ambient temperature for 24 h. GCCNP was dispersed in hydrogel precursors via sonication. The GCCNP concentrations was at 1 mg/mL in final. The hydrogel precursors were withdrawn through 5 mL medical syringes and sterilized with 290 nm UV illumination for 16 h.

### Animal

C57BL/6 wild type mice and Nrf2^−/−^ mice were purchased from The Jackson Laboratory. All mice were maintained in The University of North Carolina animal facilities, and animal experiments were conducted in accordance with the policies of the Institutional Animal Care and Use Committee (IACUC) at The University of North Carolina at Chapel Hill (UNC-CH) and the Association for Research in Vision and Ophthalmology (ARVO) statement for the use of animals in ophthalmic and vision research. All experiments were performed in compliance with ethical regulations and approved by the IACUC of UNC-CH. All the mice in experiments were 7–10 weeks postnatal, Ketamine (100 mg/kg)/Xylazine (15 mg/kg) was used for anesthesia in all cases where anesthesia was required. Euthanasia was performed using isoflurane exposure and decapitation.

### Light exposure to Nrf2^−/−^ mice

The light exposure protocol was adopted from the previous publication^37^. The Nrf2^−/−^ mice (n = 25) were dark adapted for 16 h. Next, the pupils of mice were dilated with 1% tropicamide (Bausch & Lomb) under dim red light and transferred to aluminum foil covered transparent cages under 4500 ± 300 or 10000 ± 2000 lux cold fluorescence illumination for 2 h or 3 h. The eyes of mice were imaged with Fundus camera and OCT at 1 week, 5 weeks, and 10 weeks after light exposure.

### Intravitreal injection of GCCNP and hydrogel

3 days after light exposure, 10 µL 1 mg/mL GCCNP was loaded into 10 uL nanofil syringe (World Precision instrument) with a 33 G blunt end needle. The Nrf2^−/−^ mice were anesthetized, penetrated under limbus with 27 G needle, and 1 uL GCCNP was injected via intravitreal method using micro syringe pump at 20 uL/min infusion rate. The injection of hydrogel required the same apparatus. 1 µL hydrogel precursor (with 0 or 1 mg/mL GCCNP) was injected intravitreally, 0.5 uL CaCl_2_ was infused immediately at the same infusion rate and the same position. 1 µL 0.9% PBS was injected as injection control group.

### Fundus and OCT

Mice were anesthetized and pupils were dilated with 1% tropicamide. Fundus images were captured using a Micron IV retinal imaging system (Phoenix Research Labs). Corneas were moistened with Genteal lubricant eye gel (Novartis) and positioned with the Micron eyepiece in direct contact with the eye through the gel. OCT images were captured using the full-scan setting as an average of 10 frames/scan. Three scans were taken per eye in the superior/inferior and/or nasal/temporal cardinal directions relative to the optic nerve. Retinal thickness measurements were analyzed using InSight OCT segmentation software (Voxeleron). The thickness of ONL and total retinal thickness (between and including the ganglion cell layer and the IS (inner segment)/ OS (outer segment junction)) were measured at 0.2 mm intervals from the optic nerve head (ONH)^38^.

### ERG

ERG was recorded and analyzed as suggested in previously published protocols^39^. Briefly, mice were dark-adapted overnight, and eyes were dilated with 1% tropicamide under dim red light. Genteal gel was applied to each eye, and recordings were made using gold electrodes that were placed on the cornea. The reference electrode was inserted subcutaneously behind the head, and the ground electrode in the tail. ERG was collected using an Espion E2 system (Diagnosys), and mice body temperature was maintained at 37 °C during the experiment^38^. The light stimulus proceeded with 0.1, 1, 2.25, 4, and 10 cd*s/m^2^ white light under dim light with 10 s intervals and each stimulus was recorded five times for rod cells and bipolar cells response. Next, mice were light adapted with 10 cd*s/m^2^ white light for 5 min, then proceed with 2.25 cd*s/m^2^ for cone cells response.

### Histology

The Haemotoxylin and Eosin (H&E) staining of eye tissues were carried out by the UNC Center for Gastrointestinal Biology and Disease Histology Core Facility. Briefly, fixed tissues were embedded in paraffin and sectioned into 10-mm sections for eye tissues. Sections were stained with H&E, according to conventional staining protocols, and visualized using bright-field microscopy.

### Western blotting

The retina and RPE were homogenized in 0.5 mL RIPA lysis buffer (Thermo Fisher) and heated at 70 °C for 30 min. The lysates were 1:1 mixed with Laemmli loading buffer and lysates with 20 µg protein content separated by 10% SDS-PAGE gels and wet transferred to PVDF membranes. Blots were blocked for nonspecific binding with phosphate buffer saline (PBS) with 0.1% Tween-20 (PBST) and 5% non-fat dry milk for 1 h. The membranes were incubated with 4-HNE conjugate (1:3,000), HIF-1α (1:1, 000) (Abcam), AP-1 (1:4000), and NF-κB3 (1:2000) (Proteintech) antibody diluted in PBST/5% non-fat dry milk at 40 °C overnight. After three PBST washes, the membrane was incubated with 0.1 µg/mL anti-rabbit/mouse horseradish peroxidase conjugated antibody (Abcam, 1:10, 000 dilution) in TBST/5% non-fat dry milk at ambient temperature for 1 h. The membrane was TBST washed and imaged with Bio-Rad imager with 1 mL Rio Rad Clarity substrate. Images were quantified with ImageLab software (BioRad).

### RT-PCR

The levels of IL-6, TNF-α, IFN-γ and caspase-3 obtained from homogenized retina and RPE were assessed using RT-PCR. RNA was extracted from retinas following the TRIzol protocol (Life Technologies) according to the manufacturer’s instructions. Samples were subjected to RT-PCR and normalized to β-actin to verify the efficiency of RNA extraction and for the presence of cytokines. Primers targeting the cytokines were used^32^.

1 $${\rm{Relative}}\,{\rm{Gene}}\,{\rm{Expression}}={2}^{-\Delta \Delta \mathrm{Ct}}$$

Where ΔΔCt = (Ct _Target gene, treated_ − Ct _β-actin, treated_) − (Ct _Target gene, control_ − Ct _β-actin, control_).

All reactions were performed in an ABI StepOne Plus7500 thermocycler (Life Technologies). The parameters for the amplification of pro-inflammatory cytokines were as follows: 95 °C for 2 min, 94 °C for 45 s, 56 °C for 30 s, 72 °C for 30 s for 40 cycles, and 72 °C for 5 min.

### Free radical assay

The DCF-DA and luminol assay was adopted from previous publication^16,40^. Briefly, isolated fresh retinal tissue was homogenized in 0.5 mL PBS-HEPES buffer (0.5 M PBS containing 20 mM HEPES, pH 7.2) and transferred to a 96-well plate. DCF-DA solution was added at 0.05 mM final concentration and cultured at 37 °C for 30 min. Luminol (5-amino-2,3-dihydro-1,4-phthalazinedione, Sigma–Aldrich) DMSO solution was added at 0.2 mM final concentration. Fluorescence and chemluminescence were quantified using plate reader (BMG Omega). Fluorescence intensity was measured with ex488 nm/em532 nm filter. Chemluminescience counts were obtained at 30 second intervals and the final results were represented as the area under curve (AUC) for 5 min.

### Microscopy

The imaging of cells was conducted using the Zeiss LSM-700 confocal fluorescence microscope and observer D1 fluorescence microscope with Zeiss AxioCam MRC camera. The images were processed by ZEN software.

The thickness of BrM was measured using transmission microscopy. The mice were perfused with 2.5% glutaraldehyde in cacodylate buffer (0.1 M, pH 7.4) and prepared with microscope system laboratory at UNC for post-fixation, dehydration and embedding in epoxy resin. Toluidine blue was stained at semi-thin sections (1 um), and lead citrate for ultrathin sections. BrM thickness was measured using JEOL 1230 TEM at 15000× magnification, and 10 random measurements were averaged in each sample (3 samples per group).

### Materials characterization

The absorption spectra and concentration of nanoceria and was determined using a plate reader (BMG Omega). The maximum absorption of GCCNP was determined at 295 nm and GCCNP concentration was determined using a standard curve with degraded hydrogel background subtraction. The size and zeta potential of standalone GCCNP and GCCNP released from hydrogel was determined using Nanozetasizer (Malvern).

### Statistical method

All quantitative data was expressed as the mean and standard deviation of results. The data at the endpoint was analyzed by one-way analysis of variance (ANOVA) with Tukey’s test, and time-related data were analyzed using two-way ANOVA, and a p-value less than 0.05 was considered significant.

## Supplementary information

Supplementary Materials

## References

[1] Lim, L. S., Mitchell, P., Seddon, J. M., Holz, F. G. & Wong, T. Y. Age-related macular degeneration. *The Lancet* **379**, 1728–1738 (2012). 10.1016/S0140-6736(12)60282-7 22559899

[2] Jager RD, Mieler WF, Miller JW (2008). Age-Related Macular Degeneration. N. Engl. J. Med..

[3] Pascolini D, Mariotti SP (2012). Global estimates of visual impairment: 2010. British Journal of Ophthalmology.

[4] Congdon N (2004). Causes and prevalence of visual impairment among adults in the United States. Archives of Ophthalmology.

[5] Rosenfeld PJ (2006). Ranibizumab for neovascular age-related macular degeneration. N. Engl. J. Med..

[6] Cano M (2010). Cigarette smoking, oxidative stress, the anti-oxidant response through Nrf2 signaling, and Age-related Macular Degeneration. Vision Research.

[7] Chakravarthy U (2010). Clinical risk factors for age-related macular degeneration: a systematic review and meta-analysis. BMC Ophthalmology.

[8] Jonasson F (2014). Five-Year Incidence, Progression, and Risk Factors for Age-Related Macular Degeneration: The Age, Gene/Environment Susceptibility Study. Ophthalmology.

[9] Arjamaa O, Nikinmaa M, Salminen A, Kaarniranta K (2009). Regulatory role of HIF-1α in the pathogenesis of age-related macular degeneration (AMD). Ageing Research Reviews.

[10] Chiras D, Kitsos G, Petersen MB, Skalidakis I, Kroupis C (2015). Oxidative stress in dry age-related macular degeneration and exfoliation syndrome. Critical reviews in clinical laboratory sciences.

[11] Evans J (2008). Antioxidant supplements to prevent or slow down the progression of AMD: a systematic review and meta-analysis. Eye.

[12] Evans JB, Syed BA (2013). New hope for dry AMD?. Nat Rev Drug Discov.

[13] Holz FG, Schmitz-Valckenberg S, Fleckenstein M (2014). Recent developments in the treatment of age-related macular degeneration. The Journal of Clinical Investigation.

[14] Fleckenstein M (2018). The Progression of Geographic Atrophy Secondary to Age-Related Macular Degeneration. Ophthalmology.

[15] Karakoti AS (2008). Nanoceria as Antioxidant: Synthesis and Biomedical Applications. JOM (Warrendale, Pa.: 1989).

[16] Mitra RN (2017). Glycol Chitosan Engineered Autoregenerative Antioxidant Significantly Attenuates Pathological Damages in Models of Age-Related Macular Degeneration. ACS Nano.

[17] Hirst SM (2009). Anti-inflammatory Properties of Cerium Oxide Nanoparticles. Small.

[18] Grulke E (2014). Nanoceria: factors affecting its pro- and anti-oxidant properties. Environmental Science: Nano.

[19] Cai X, Seal S, McGinnis JF (2014). Sustained inhibition of neovascularization in vldlr^−/−^ mice following intravitreal injection of cerium oxide nanoparticles and the role of the ASK1-P38/JNK-NF-kappaB pathway. Biomaterials.

[20] Wang K, Nune KC, Misra RDK (2016). The functional response of alginate-gelatin-nanocrystalline cellulose injectable hydrogels toward delivery of cells and bioactive molecules. Acta Biomaterialia.

[21] Cai, X. & McGinnis, J. F. Nanoceria: a Potential Therapeutic for Dry AMD. In *Retinal Degenerative Diseases: Mechanisms and Experimental Therapy* (eds. Bowes Rickman, C.*, et al*.) 111–118 (Springer International Publishing, Cham, 2016). 10.1007/978-3-319-17121-0_16 26427401

[22] Pujol-Lereis, L. M., Schäfer, N., Kuhn, L. B., Rohrer, B. & Pauly, D. Interrelation between oxidative stress and complement activation in models of age-related macular degeneration. in *Retinal Degenerative Diseases* 87–93 (Springer, 2016). 10.1007/978-3-319-17121-0_13 26427398

[23] Zhao Z (2011). Age-Related Retinopathy in NRF2-Deficient Mice. PLoS ONE.

[24] Rozanowska MB (2012). Light-induced damage to the retina: current understanding of the mechanisms and unresolved questions: a symposium-in-print. Photochemistry and photobiology.

[25] Imamura Y (2006). Drusen, choroidal neovascularization, and retinal pigment epithelium dysfunction in SOD1-deficient mice: A model of age-related macular degeneration. Proceedings of the National Academy of Sciences of the United States of America.

[26] Nagar S (2017). MEF2D haploinsufficiency downregulates the NRF2 pathway and renders photoreceptors susceptible to light-induced oxidative stress. Proc Natl Acad Sci USA.

[27] Sarks S, Cherepanoff S, Killingsworth M, Sarks J (2007). Relationship of Basal laminar deposit and membranous debris to the clinical presentation of early age-related macular degeneration. Invest Ophthalmol Vis Sci.

[28] Kai, W., Narayan, M. R., Min, Z. & Zongchao, H. Nanoceria‐loaded injectable hydrogels for potential age‐related macular degeneration treatment. *Journal of Biomedical Materials Research Part A* **106**, 2795–2804 (2018). 10.1002/jbm.a.36450 PMC6231995 29752862

[29] Wenzel A (2001). Prevention of Photoreceptor Apoptosis by Activation of the Glucocorticoid Receptor. Investigative Ophthalmology & Visual Science.

[30] Sæther HV, Holme HK, Maurstad G, Smidsrød O, Stokke BT (2008). Polyelectrolyte complex formation using alginate and chitosan. Carbohydrate Polymers.

[31] Kanagasingam Y (2014). Progress on retinal image analysis for age related macular degeneration. Progress in Retinal and Eye Research.

[32] Kauppinen A (2012). Oxidative stress activates NLRP3 inflammasomes in ARPE-19 cells—Implications for age-related macular degeneration (AMD). Immunology Letters.

[33] Sasaki M (2009). Neuroprotective Effect of an Antioxidant, Lutein, during Retinal Inflammation. Investigative Ophthalmology & Visual Science.

[34] Käsdorf BT, Arends F, Lieleg O (2015). Diffusion Regulation in the Vitreous Humor. Biophysical Journal.

[35] Tomida H (2010). Polysaccharides as potential antioxidative compounds for extended-release matrix tablets. Carbohydrate Research.

[36] Kowluru RA, Chan P-S (2007). Oxidative stress and diabetic retinopathy. Exp Diabetes Res.

[37] Grimm, C. & Remé, C. E. Light damage as a model of retinal degeneration. In *Retinal Degeneration* 87–97 (Springer, 2012). 10.1007/978-1-62703-080-9_6 23150362

[38] Liang KJ (2017). AAV-Nrf2 promotes protection and recovery in animal models of oxidative stress. Molecular Therapy.

[39] McCulloch DL (2015). ISCEV Standard for full-field clinical electroretinography (2015 update). Documenta ophthalmologica. Advances in ophthalmology.

[40] Fang I-M, Yang C-M, Yang C-H, Chiou S-H, Chen M-S (2013). Transplantation of induced pluripotent stem cells without C-Myc attenuates retinal ischemia and reperfusion injury in rats. Experimental eye research.

