# Supplementary material for: Light-induced Nrf2−/− mice as atrophic age-related macular degeneration model and treatment with nanoceria laden injectable hydrogel
Source: Sci Rep. 2019 Oct 10;9:14573. doi: 10.1038/s41598-019-51151-7 (PMC6787253; doi:10.1038/s41598-019-51151-7)
Supplement: Supplementary file 1 — Supplementary Materials [file 41598_2019_51151_MOESM1_ESM.docx]

**Light-induced Nrf2^-/-^ mice as atrophic age-related macular degeneration model and treatment with nanoceria laden injectable hydrogel**

Kai Wang1, Min Zheng1, Kaitlyn Lee Lester1, Zongchao Han1, 2, 3

^1^ Department of Ophthalmology, University of North Carolina at Chapel Hill, Chapel Hill, NC 27599 USA

^2^ Carolina Institute for Nano Medicine, University of North Carolina at Chapel Hill, Chapel Hill, NC 27599 USA

^3^ Division of Pharmacoengineering & Molecular Pharmaceutics, Eshelman School of Pharmacy, University of North Carolina at Chapel Hill, Chapel Hill, NC 27599 USA

Table 1. Luminol free radical assay results of wild type mice, naïve Nrf2^-/-^ mice, 2 h light exposed Nrf2^-/-^ mice, 3 h light exposed Nrf2^-/-^ mice, GCCNP treated 3 h light exposed Nrf2^-/-^ mice, and GCCNP-laden hydrogel treated 3 h light exposed Nrf2^-/-^ mice at PI-10 weeks.

| Group | Luminescence (AUC*1000) |
| --- | --- |
| **Wild Type (C57BL/6)** | 80±7 |
| **Nrf2^-/-^ Naive** | 87±22 |
| **Nrf2^-/-^ Light exposure (2h)** | 210±65 |
| **Nrf2^-/-^ Light exposure (3h)** | 330±20 |
| **Nrf2^-/-^ light exposure (3h) and GCCNP laden hydrogel injected** | 51±10 |
| **Nrf2^-/-^ light exposure (3h) and GCCNP injected** | 121±15 |


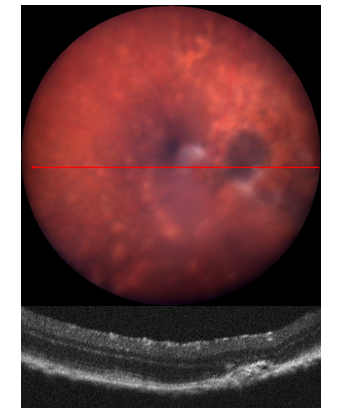


Figure S1. Fundus and OCT images of 4500lux 3h light exposed Nrf2^-/-^ mice 16 weeks post-light exposure. Advanced stage dry AMD phenotypes were found from Fundus and OCT images.


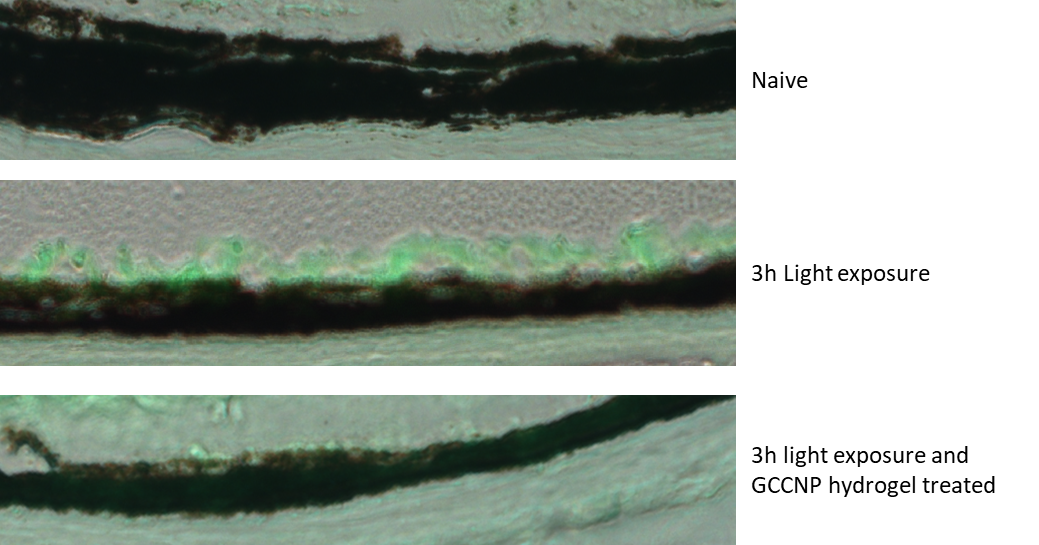


Figure S2. Green autofluorescence was observed from lipofuscin accumulation. The autofluorescence was diminished on native and GCCNP laden hydrogel treated mice.


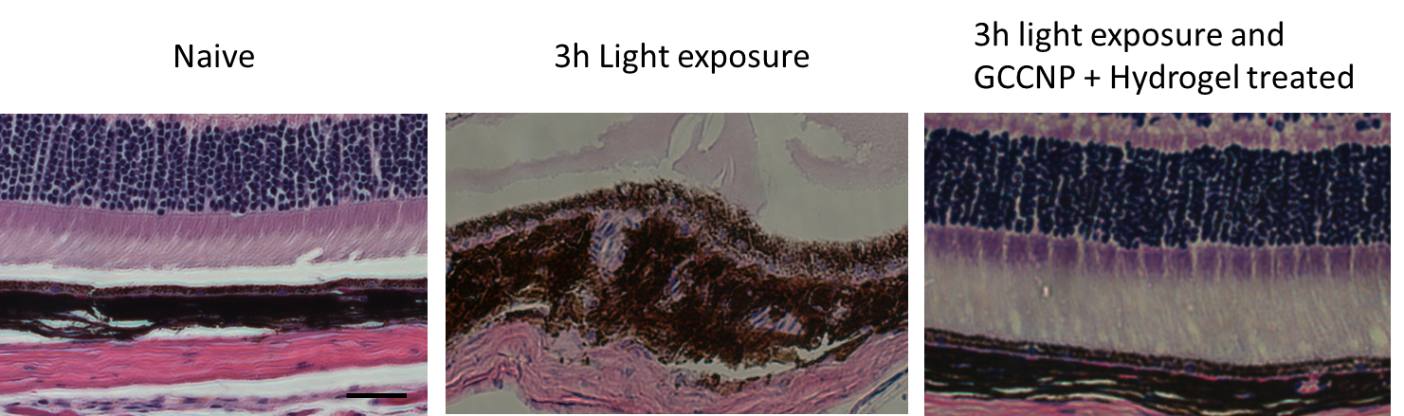


Figure S3. Histology section of choroid layers showed choroid layer thickness increased at PI-10 weeks after 3 h light exposure. The GCCNP laden hydrogel treated mice have a choroid layer thickness similar to that of naïve Nrf2^-/-^ mice.


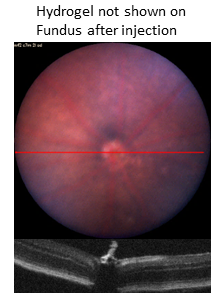


Figure S4. Fundus and OCT images of GCCNP laden hydrogel inject intravitreally on native

Nrf2^-/-^ mice. The hydrogel was not shown on Fundus and no signs of retinal toxicity was observed.


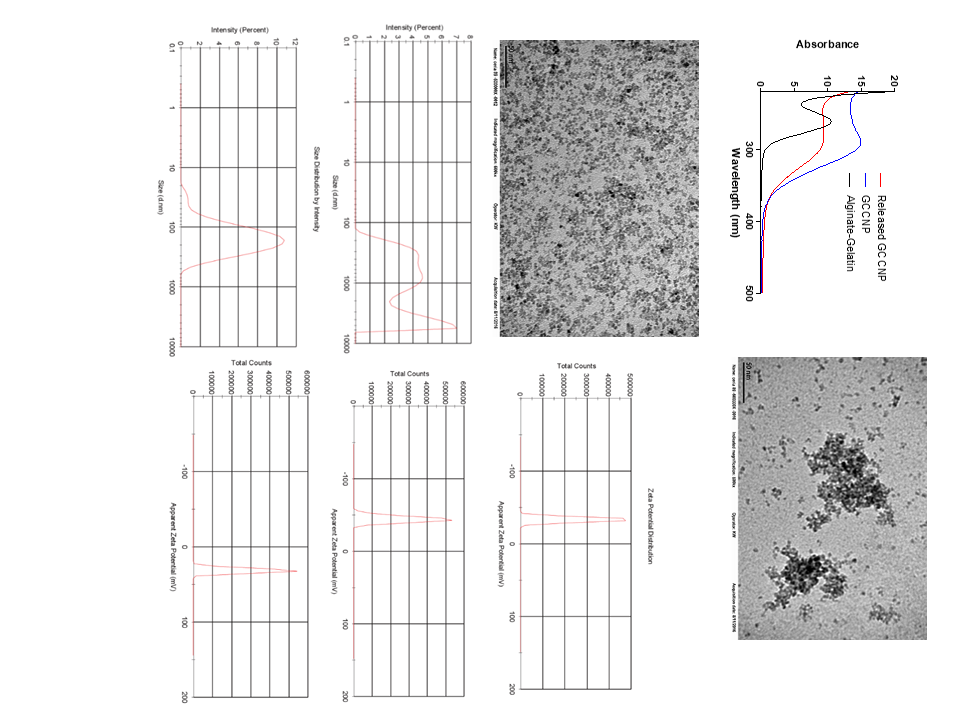


Figure S5. UV-Vis spectra of GCCNP, GCCNP released from hydrogel, and alginate-gelatin (upper left panel), TEM images of GCCNP, GCCNP released from hydrogel (upper right and second left panel), Dynamic light scattering size of GCCNP and GCCNP released from hydrogel (second right panel and third panel), and Zeta potential of GCCNP, GCCNP released from hydrogel, and alginate-gelatin.


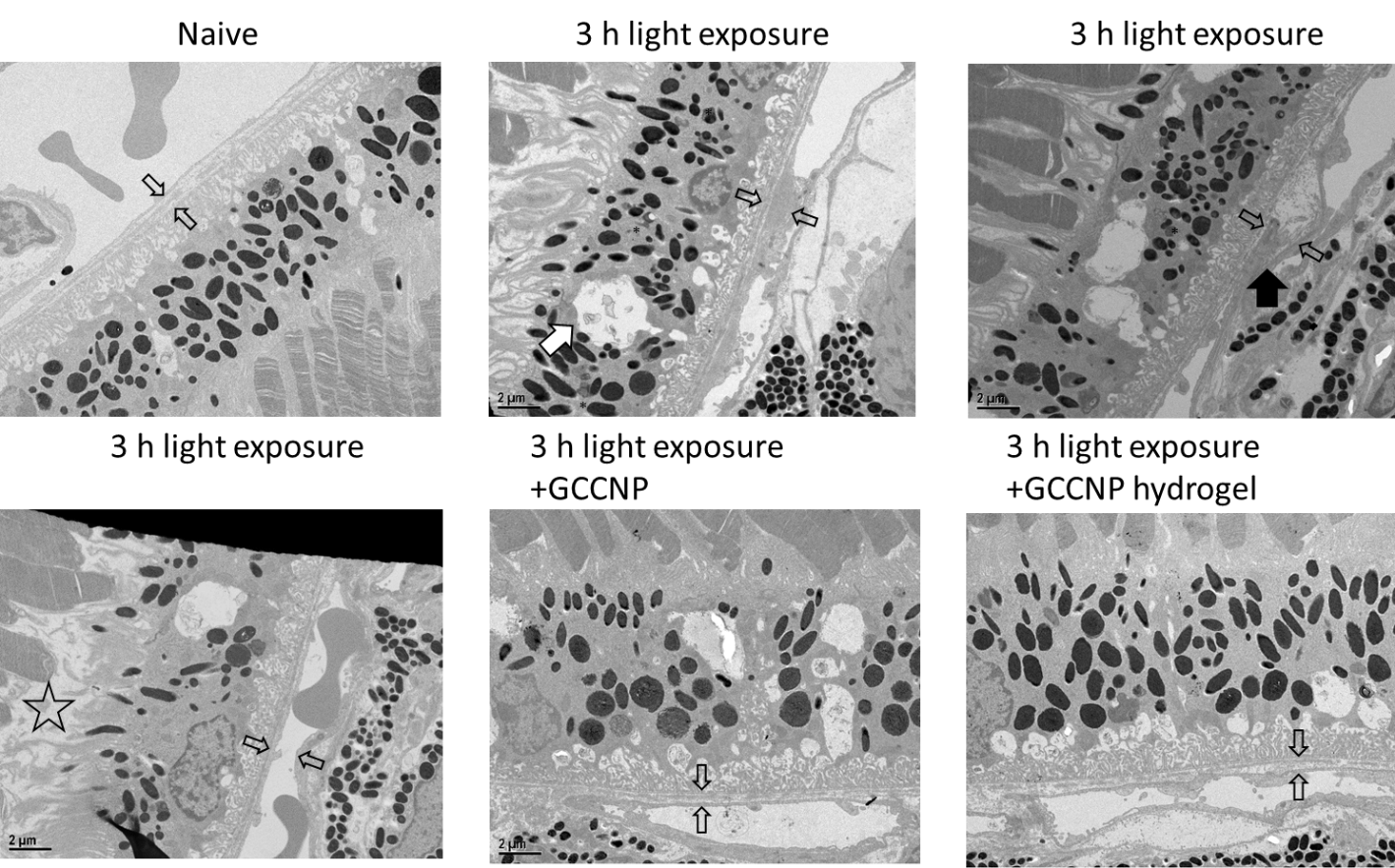


Figure S6. The original TEM images of RPE and PRC of naïve, 3 h 4500 lux light-exposed Nrf2^-/-^ mice without treatment, with 1 µg GCCNP or 1µg GCCNP in 1 µL hydrogel by intravitreal injection at 10 weeks post-injection. (White Arrow: vacuole, black arrow: drusen-like deposit, asterisk: lipofuscin granule, hollowed arrow: Burch’s membrane, and star-shape: PRC cells detachment)


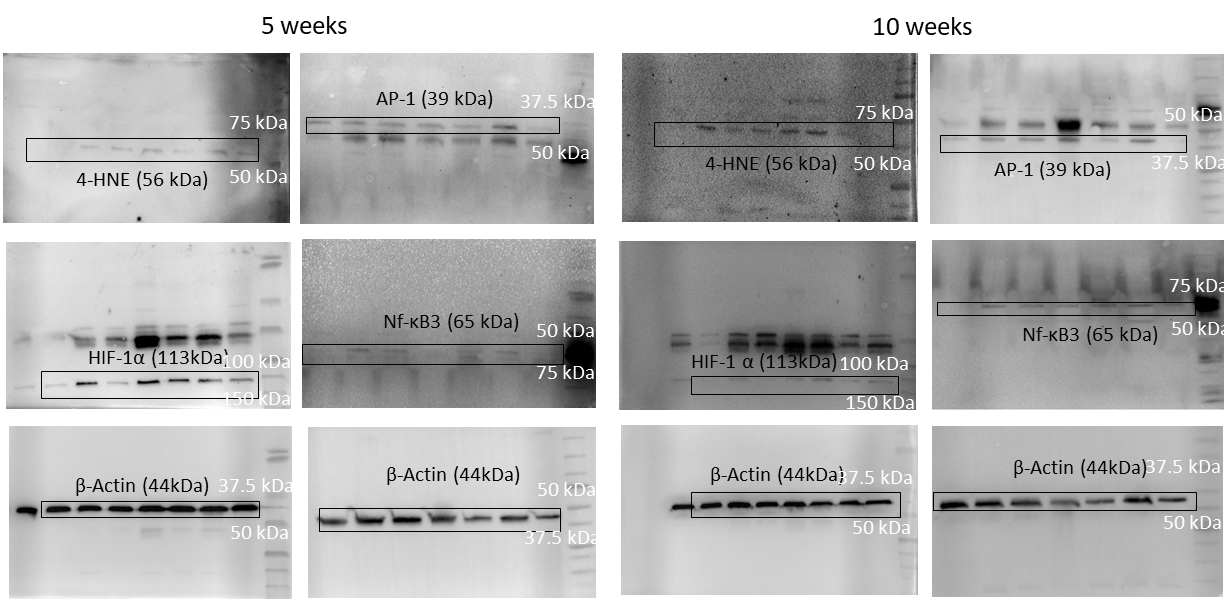


Figure S7. Original western blotting of 4-HNE, HIF-1α, AP-1, and Nf-kB3. Band from right to left: Naïve Nrf2^-/-^ mice, 2 h light-exposed Nrf2^-/-^ mice,3 h light-exposed Nrf2^-/-^ mice, 3 h light-exposed Nrf2^-/-^ mice treated with PBS, 3 h light-exposed Nrf2^-/-^ mice by 1 µg GCCNP, or 3 h light-exposed Nrf2^-/-^ mice 1µg GCCNP in 1 µL hydrogel by intravitreal injection at 5 weeks and 10 weeks.
